# Influence of time to ablation on outcomes among patients with atrial fibrillation with pre-existing heart failure

**DOI:** 10.1016/j.hroo.2024.07.016

**Published:** 2024-08-05

**Authors:** Adi Lador, Sonia Maccioni, Rahul Khanna, Dongyu Zhang

**Affiliations:** ∗ Division of Cardiac Electrophysiology, Department of Cardiology, Houston Methodist DeBakey Heart and Vascular Center, Houston Methodist Hospital, Houston, Texas; † Franchise Health Economics and Market Access, Johnson & Johnson MedTech, Irvine, California; ‡ MedTech Epidemiology and Real-World Data Sciences, Johnson & Johnson, New Brunswick, New Jersey

**Keywords:** Atrial fibrillation, Heart failure, Catheter ablation, Epidemiology, Cardiology

## Abstract

**Background:**

Atrial fibrillation (AF) and heart failure (HF) are cardiac disorders that often coexist.

**Objective:**

This study aimed to investigate how time to ablation could influence the outcomes of AF patients with pre-existing HF.

**Methods:**

Using the 2013 to 2022 Optum Clinformatics database, AF patients with pre-existing HF were classified into 2 groups: early ablation (ablation within 6 months of AF diagnosis) and late ablation (ablation in the 6- to 24-month period after AF diagnosis). Outcomes including AF-related hospitalization, electrical cardioversion, repeat ablation, antiarrhythmic drug (AAD) use, and AF recurrence (a composite outcome of the aforementioned events) were assessed in the postblanking 24-month period. Inverse probability of treatment weighted Poisson regression estimated risk ratio (RR) and 95% confidence interval (CI) for each outcome.

**Results:**

Overall, 601 patients were identified (early ablation: 347; late ablation: 254). In 24 months, the weighted data suggested that patients in the early ablation cohort had significantly lower rate of composite outcome (49.32% vs 61.39%, *P =* .01), repeat ablation (8.56% vs 17.35%, *P <* .01), and AAD use (35.95% vs 47.92%, *P =* .01). Early ablation was associated with a 20%, 51%, and 25% lower risk of composite outcome (RR 0.80, 95% CI 0.69–0.94), repeat ablation (RR 0.49, 95% CI 0.31–0.79), and AAD use (RR 0.75, 95% CI 0.61–0.92), respectively. No significant difference in AF-related hospitalization and electrical cardioversion were observed.

**Conclusion:**

AF patients with pre-existing HF undergoing ablation within 6 months of AF diagnosis have a lower risk of AF recurrence than those undergoing late ablation, which was evidenced by a lower rate of repeat ablation and AAD use.

## Graphical abstract

**Figure:**
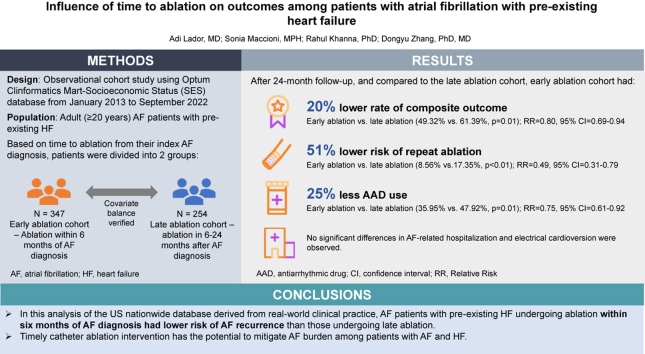

**Key Findings**

▪ In this retrospective analysis of atrial fibrillation (AF) patients with pre-existing heart failure (HF) using a nationally representative administrative claims database, patients undergoing catheter ablation (CA) within 6 months of AF diagnosis had a lower 24- and 12-month risk of AF recurrence than those receiving CA in 6 to 24 months of AF diagnosis.
▪ In this study, the difference of AF recurrence risk by timing of CA is consistent by sex.
▪ The lower risk of AF recurrence associated with early CA is primarily evidenced by a lower risk of repeat CA and antiarrhythmic drug use.

## Introduction

Atrial fibrillation (AF) and heart failure (HF) are both common cardiac disorders associated with poor prognosis.^1^, ^2^, ^3^, ^4^ These conditions often coexist, they share some similar etiological characteristics, and one potentially can lead to the other.^5^^,^^6^ In clinical practice, pre-existing HF may induce complexity in management and care delivery for patients diagnosed with AF,^2^^,^^7^, ^8^, ^9^ making AF patients with HF encounter more challenges in care than AF patients without HF. In patients with HF, AF is present in 10% to 60% of the cases.^6^^,^^9^^,^^10^

Catheter ablation (CA) has been widely used to treat AF, and the current guidelines recommend CA in patients with AF and HF who remain symptomatic on medical therapy.^8^^,^^11^, ^12^, ^13^ However, time to ablation could have an influence on the outcomes. D'Angelo and colleagues^14^ showed that patients with AF undergoing ablation within 6 months of diagnosis had lower healthcare utilization compared with patients who underwent ablation between 6 and 12 months of diagnosis. Similarly, patients with AF and preserved ejection fraction (EF) who underwent CA within 180 days of AF diagnosis had lower 1-year recurrence rates than those who underwent CA after 180 days.^15^ However, both of these studies included only a small proportion of patients with HF (12%–19%). Based on results of EAST-AFNET 4 (Early Treatment of Atrial Fibrillation for Stroke Prevention Trial 4) trial, the 2023 American College of Cardiology/American Heart Association/American College of Clinical Pharmacology/Heart Rhythm Society guidelines suggest that early rhythm control (within 1 year of AF diagnosis) can be useful to reduce risk of stroke, hospitalization, and death (Class of Recommendation: IIa).^16^ However, very early ablation (within 1 month of AF diagnosis), compared with ablation within 1 year of AF diagnosis, was not associated with improved outcomes in a multicenter randomized controlled trial (9% had HF).^17^ To date, there is limited research examining the impact of time to ablation among AF patients with pre-existing HF.

Depicting the outcomes among incident AF patients with pre-existing HF by timing of CA receipt can help clinicians better understand the role of timing of CA in these patients. In this study, we examine the association between time to ablation and outcomes among AF patients with preexisting HF.

## Methods

### Data source

The Optum Clinformatics Data Mart–Socioeconomic Status (SES) Database between January 2013 and September 2022 was used for study purposes. The Optum-SES database is a deidentified administrative claims database which contains information from commercially insured individuals or individuals with Medicare Advantage health plans. This database has inpatient, outpatient, and pharmacy claims from approximately 18 million covered lives annually.^18^ In addition, the Optum-SES database contains information on SES (eg, education and income) and location for individuals with both medical and pharmacy coverage at the U.S. Census Division level.^19^ This analysis of the Optum Clinformatics database was conducted under an exemption from Institutional Review Board oversight for U.S.-based studies using de-identified healthcare records, as dictated by Title 45 Code of Federal Regulations (45 CFR 46.101(b)(4)) (https://www.govinfo.gov/content/pkg/CFR-2011-title45-vol1/pdf/CFR-2011-title45-vol1.pdf).

### Study population

**Figure 1:**
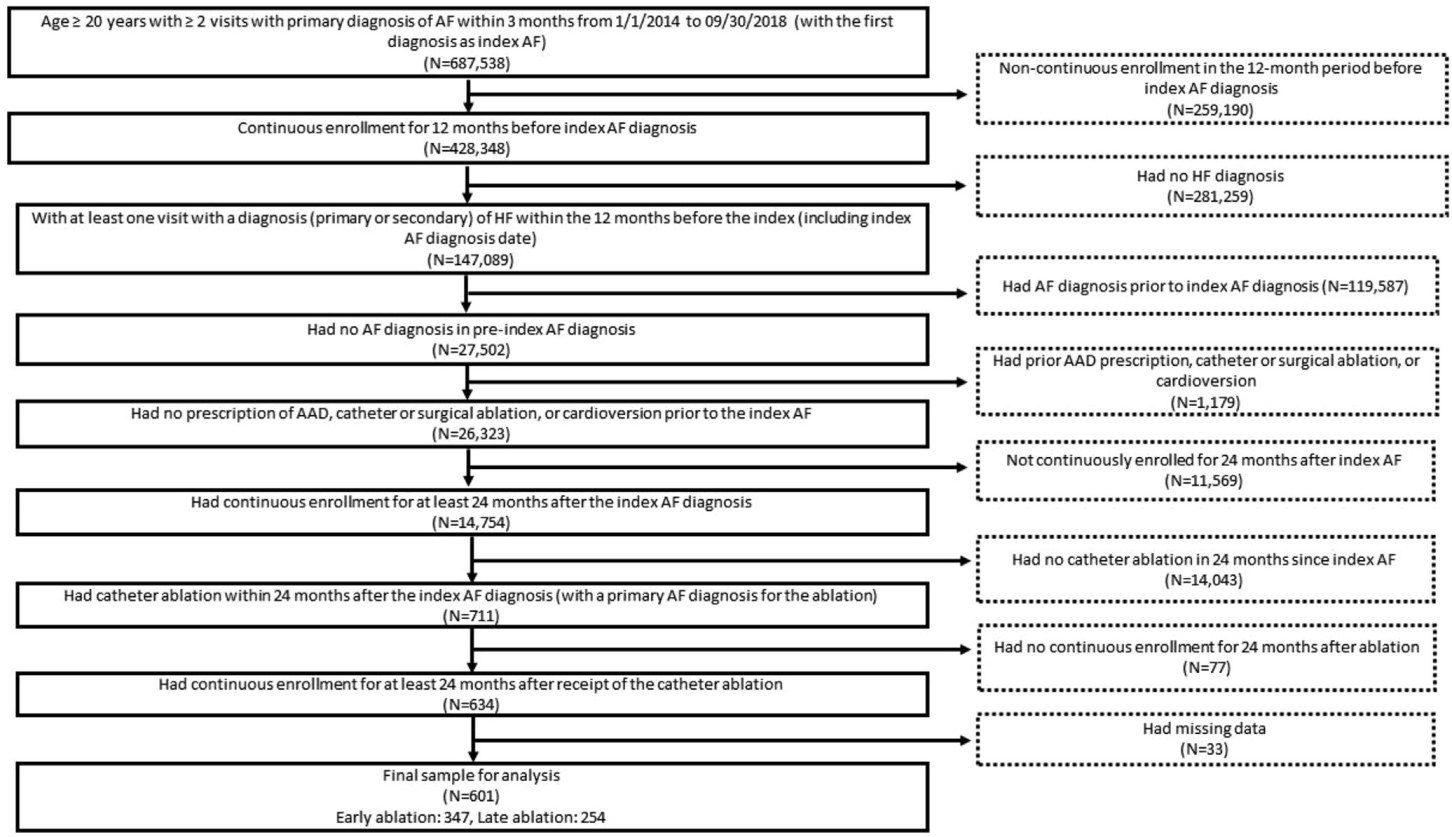
Flowchart of participants selection. Early ablation was defined as ablation within 6 months after index atrial fibrillation (AF) diagnosis. Late ablation was defined as ablation 6–24 months after the index AF diagnosis. AAD = antiarrhythmic drug; HF = heart failure.

Patients (≥20 years of age) who had at least 2 medical services visits with primary diagnoses of AF (based on International Classification of Diseases–Ninth Revision and International Classification of Diseases–Tenth Revision) within 3 months between January 1, 2014, and September 30, 2018, were identified; the date of index AF diagnosis was defined as the date of the first of these 2 claims. We did not differentiate between inpatient and outpatient visits when identifying patients with AF. These patients were then included for analysis if they met the following criteria: (1) had continuous enrollment for at least 12 months before the index AF diagnosis; (2) had at least 1 prior medical visit with a diagnosis of HF in the 12 months prior to or during index AF diagnosis; (3) had no prior diagnosis of AF within the 12 months before the index AF diagnosis; (4) had no prescription of antiarrhythmic drug (AAD), catheter or surgical ablation, or cardioversion in the 12 months prior to the index AF diagnosis; (5) had continuous enrollment for at least 24 months since the index AF diagnosis; (6) had CA (with primary diagnosis of AF for the ablation) within 24 months since the index AF diagnosis; (7) had continuous enrollment for at least 24 months after the receipt of CA; and (8) had no missing data of other covariates. Ascertainment of CA was based on Current Procedural Terminology, International Classification of Diseases–Tenth Revision codes, and/or International Classification of Diseases–Ninth Revision code. Based on time to ablation from their index AF diagnosis, the final sample of patients was classified into 2 groups: an early ablation cohort (ie, those who had ablation within 6 months of index AF diagnosis) and a late ablation cohort (ie, those who had ablation in the 6- to 24-month period post–index AF diagnosis). Figure 1 depicts the study inclusion and exclusion criteria.

### Study outcome

A 3-month postablation blanking period was applied in the study.^20^ Only events occurring after the blanking period were treated as the study outcome in analysis. Study outcomes included AF-related hospitalization, electrical cardioversion, repeat CA, AAD use, and a composite outcome of the aforementioned events assessed in the postblanking 24-month period. A primary diagnosis of AF was required for AF-related hospitalization, electrical cardioversion, and repeat CA. Subgroup analysis by sex was performed for the examination of postblanking 24-month period outcomes. As part of sensitivity assessment, the aforementioned events were assessed for the overall sample in the postblanking 12-month period after index ablation.

### Study covariates

Patients’ sociodemographics including age, sex, race and ethnicity, education, annual household income, and region were assessed. Race and ethnicity were categorized as non-Hispanic White, non-Hispanic Black, Hispanic, Asian, and Other. Geographic regions were categorized based on the U.S. Census Bureau regions: Midwest, Northeast, South, and West.^21^ Education was categorized as without a bachelor degree and bachelor or higher; annual household income was categorized as <$50,000, $50,000 to $99,999, and ≥$100,000.^22^ Patients’ clinical characteristics included Elixhauser Comorbidity Index and CHA_2_DS_2_-VASc (congestive heart failure, hypertension, age ≥75 years, diabetes mellitus, prior stroke or transient ischemic attack or thromboembolism, vascular disease, age 65-74 years, sex category). Subtypes of AF (paroxysmal, persistent, other) and HF (diastolic, systolic, diastolic and systolic, other) were assessed by International Classification of Diseases codes. HF-related health conditions (chest pain, edema, orthopnea, and ascites), lifestyle behaviors associated with HF (alcohol abuse and smoking), medications to control HF (angiotensin-converting enzyme inhibitor, beta-blocker, angiotensin receptor blocker, diuretic, digoxin), and HF-related hospitalization and emergency room visit, as well as history of utilization of cardiac resynchronization therapy defibrillator and/or implantable cardioverter-defibrillator, were used to reflect severity of HF. Supplemental Table 1 includes relevant codes for the assessment of study covariates.

### Statistical analysis

Propensity score (PS) was estimated based on the previously mentioned covariates. To mitigate bias due to covariate measurement error, we applied trimming to identify patients with PS between 0.10 and 0.90,^23^^,^^24^ and we generated a post-trimming PS among these patients. Inverse probability of treatment weighting (IPTW)^25^ was applied to reduce impact of confounding; specifically, numerator of the weight was the probability of actual treatment (early or late ablation), and the denominator was post-trimming PS and 1 – PS for the early and late ablation groups, respectively. We descriptively summarized distributions of study covariates for samples before and after IPTW. Standardized mean difference (SMD) was used to assess if distributions of study covariates were well balanced between the early and late ablation groups, with a |SMD|<0.10 indicating a well-balanced distribution.^26^

The rate of each study outcome was summarized for samples before and after IPTW, and we used chi-square tests to assess if their rates differed by timing of ablation. After IPTW, we used Poisson regression to estimate the risk ratio (RR) and 95% confidence interval (CI) for each outcome, using late ablation as the reference group in the model. To explore if the difference in risk of outcomes between treatment modalities (early vs late ablation) varied by sex, we applied the Poisson regression for female and male patients, respectively. In subgroup analysis, we applied the same methods of PS estimation, trimming, and weighting for each subpopulation (female vs male) before estimating the RR.

Two-sided values of *P <* .05 were considered to be statistically significant. Statistical analyses were conducted with R software (version 4.1.2; R Foundation for Statistical Computing).

## Results

After applying study criteria, 601 patients were identified. Among these patients, 347 were classified as the early ablation cohort and 254 as the late ablation cohort. Distributions of covariates of the study population are listed in Table 1. Before IPTW, patients in the early ablation group (mean age 69.82 ± 9.93 years) were slightly older than patients in the late ablation group (mean age 68.53 ± 8.95 years). In both groups, over half of the patients were male (early: 56.77%, late: 57.87%). The early ablation group had a slightly higher proportion of non-White patients. Significant differences were observed in an unweighted population on other study covariates including education, income, region, Elixhauser Comorbidity Index, AF subtype, ascites, beta-blocker and digoxin utilization, sleep apnea, congenital heart disease, hypertension, obesity, anemia, and history of cardiac resynchronization therapy defibrillator and/or implantable cardioverter-defibrillator. Based on the SMD estimated after IPTW, all the study covariates were well balanced in the early and late ablation groups after IPTW (Table 1).

**Table 1:** Study characteristics of study population

	Unweighted		SMD	Weighted∗		SMD
	Early ablation (n = 347)	Late ablation (n = 254)		Early ablation	Late ablation	
Age	69.82 ± 9.93	68.53 ± 8.95	0.136	69.48 ± 9.48	69.24 ± 8.83	0.026
<65 y	91 (26.22)	63 (24.80)	0.234	24.70	23.97	0.024
65–74 y	141 (40.63)	130 (51.18)		46.76	47.94	
≥75 y	115 (33.14)	61 (24.02)		28.55	28.09	
Sex						
Female	150 (43.23)	107 (42.13)	0.022	42.96	42.59	0.008
Male	197 (56.77)	147 (57.87)		57.04	57.41	
Race/ethnicity						
NHW	279 (80.40)	214 (84.25)	0.183	82.05	82.45	0.030
NHB	27 (7.78)	18 (7.09)		7.81	7.35	
Hispanic	31 (8.93)	12 (4.72)		6.87	6.65	
Asian	4 (1.15)	3 (1.18)		1.30	1.57	
Other	6 (1.73)	7 (2.76)		1.97	1.98	
Education						
Without a bachelor’s degree	280 (80.69)	220 (86.61)	0.161	83.69	83.38	0.008
Bachelor’s degree or higher	67 (19.31)	34 (13.39)		16.31	16.62	
Annual household income						
<$50,000	132 (38.04)	84 (33.07)	0.109	35.74	35.79	0.002
$50,000–$99,999	130 (37.46)	106 (41.73)		39.43	39.47	
≥$100,000	85 (24.50)	64 (25.20)		24.83	24.75	
Geographic region						
Midwest	82 (23.63)	72 (28.35)	0.197	24.84	23.84	0.028
Northeast	32 (9.22)	28 (11.02)		9.69	9.62	
South	159 (45.82)	92 (36.22)		43.09	44.31	
West	74 (21.33)	62 (24.41)		22.38	22.22	
Elixhauser Comorbidity Index						
≤3	38 (10.95)	29 (11.42)	0.102	11.35	11.31	0.003
4–6	176 (50.72)	140 (55.12)		52.80	52.94	
≥7	133 (38.33)	85 (33.46)		35.85	35.75	
CHA_2_DS_2_-VASc						
≤3	125 (36.02)	93 (36.61)	0.093	36.94	37.53	0.014
4–5	154 (44.38)	120 (47.24)		45.36	45.17	
≥6	68 (19.60)	41 (16.14)		17.70	17.31	
AF subtype						
Paroxysmal	98 (28.24)	76 (29.92)	0.268	29.10	28.39	0.054
Persistent	61 (17.58)	22 (8.66)		13.87	15.80	
Other	188 (54.18)	156 (61.42)		57.03	55.81	
HF subtype						
Diastolic	88 (25.36)	54 (21.26)	0.099	24.33	25.78	0.047
Systolic	117 (33.72)	89 (35.04)		35.45	36.00	
Diastolic and systolic	44 (12.68)	33 (12.99)		11.67	10.65	
Other	98 (28.24)	78 (30.71)		28.55	27.57	
HF-related condition						
Chest pain	156 (44.96)	119 (46.85)	0.038	45.53	44.58	0.019
Edema	76 (21.90)	65 (25.59)	0.087	23.39	22.94	0.011
Orthopnea	4 (1.15)	2 (0.79)	0.037	1.06	1.05	0.001
Ascites	7 (2.02)	1 (0.39)	0.149	0.83	0.65	0.021
HF-related medication						
ACE inhibitor	83 (23.92)	67 (26.38)	0.057	24.86	24.67	0.004
Beta-blockers	154 (44.38)	95 (37.40)	0.142	41.57	40.26	0.027
ARB	63 (18.16)	40 (15.75)	0.064	17.35	16.21	0.030
Diuretics	126 (36.31)	96 (37.80)	0.031	36.61	36.23	0.008
Digoxin	4 (1.15)	7 (2.76)	0.116	2.24	1.95	0.020
HF-related lifestyle risk factor						
Alcohol abuse	20 (5.76)	12 (4.72)	0.047	5.29	5.77	0.021
Smoking	61 (17.58)	48 (18.90)	0.034	17.95	17.76	0.005
Coexisting health conditions						
Sleep apnea	67 (19.31)	69 (27.17)	0.187	22.41	22.81	0.010
Coronary artery disease	99 (38.98)	136 (39.19)	0.004	38.98	39.45	0.010
Congenital heart disease	11 (3.17)	4 (1.57)	0.105	2.30	1.95	0.024
Hypertension	299 (86.17)	208 (81.89)	0.117	83.81	83.58	0.006
Diabetes	126 (36.31)	93 (36.61)	0.006	35.93	35.09	0.017
Obesity	114 (32.85)	106 (41.73)	0.184	36.95	36.23	0.015
Chronic pulmonary disease	119 (34.29)	88 (34.65)	0.007	33.77	35.40	0.034
Anemia	28 (8.07)	14 (5.51)	0.102	6.80	7.07	0.011
HF-related hospitalization	79 (22.77)	53 (20.87)	0.046	21.71	21.57	0.004
HF-related ER visit	18 (5.19)	14 (5.51)	0.014	5.42	5.50	0.004
CRT-D/ICD	30 (8.65)	11 (4.33)	0.176	5.98	4.97	0.044

Values are mean ± SD or n (%).Early ablation was defined as ablation within 6 months after index AF diagnosis. Late ablation was defined as ablation 6–24 months after the index AF diagnosis.

ACE = angiotensin-converting enzyme; ARB = angiotensin receptor blockers; CHA_2_DS_2_-VASc = congestive heart failure, hypertension, age ≥75 years, diabetes mellitus, prior stroke or transient ischemic attack or thromboembolism, vascular disease, age 65–74 years, sex category; CRT-D = cardiac resynchronization therapy defibrillator; HF = heart failure; ICD = implantable cardioverter-defibrillator; NHB = non-Hispanic Black; NHW = non-Hispanic White; SMD = standardized mean difference.

∗ Trimming was applied, and we only included patients with propensity score between 0.10 and 0.90 for inverse probability of treatment weighting. Specifically, for post-trimming sample, the weight is a marginal probability of early ablation×(1/propensity score) for early ablation group and marginal probability of late ablation×[1/(1 – propensity score)] for late ablation group.

**Figure 2:**
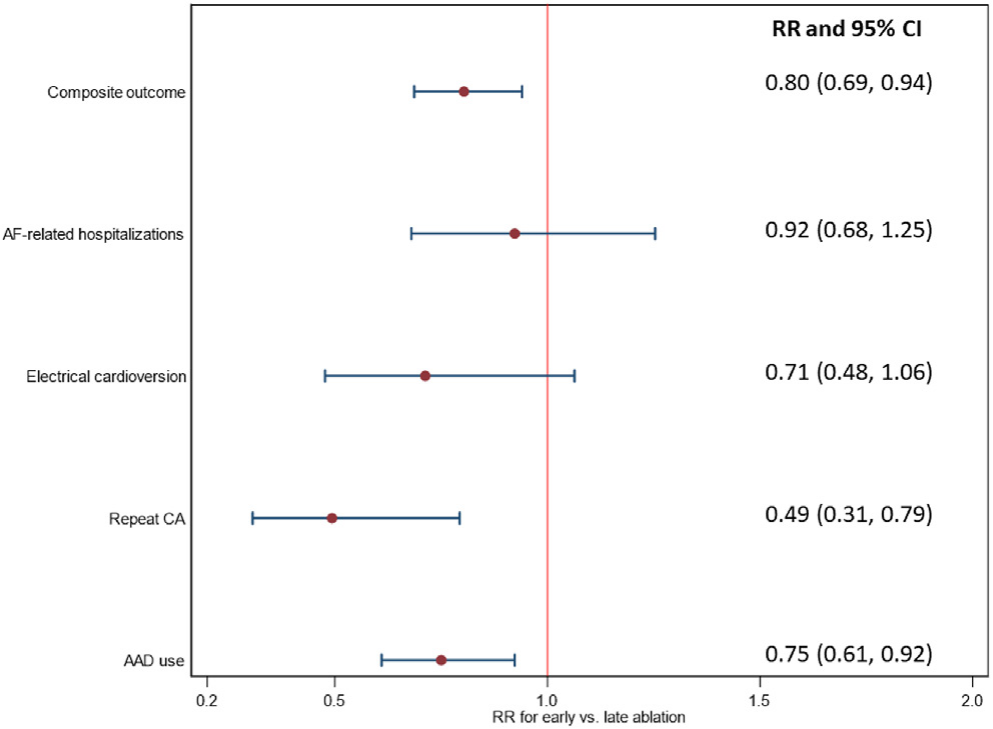
Analysis for association between time to ablation (early vs late) and outcomes within 24 months after catheter ablation (CA). The first 90 days after CA receipt was blanking period, and events that occurred within this period were not treated as the outcome of interest for analysis. Early ablation was defined as ablation within 6 months after index atrial fibrillation (AF) diagnosis. Late ablation was defined as ablation 6 to 24 months after the index AF diagnosis. AAD = antiarrhythmic drug; CI = confidence interval; RR = risk ratio.

When investigating the 24-month outcome, chi-square tests indicated that rate of the composite outcome (unweighted: 48.10% vs 62.41%, *P <* .01; weighted: 49.32% vs 61.39%, *P =* .01), repeat CA (unweighted: 8.15% vs 16.92%, *P <* .01; weighted: 8.56% vs 17.35%, *P <* .01), and AAD use (unweighted: 33.97% vs 49.25%, *P <* .01; weighted: 35.95% vs 47.92%, *P =* .01) were significantly lower in the early ablation group for both the unweighted and weighted samples (Table 2). When examining the 24-month outcome using Poisson regression models, results suggested that patients in the early ablation group had a 20% lower risk of composite outcome than the late ablation group (RR 0.80, 95% CI 0.69–0.94). No significant differences in AF-related hospitalization (RR 0.92, 95% CI 0.68–1.25) and electrical cardioversion (RR 0.71, 95% CI 0.48–1.06) were observed among the early and late ablation cohorts in the overall sample. However, the risk of repeat CA (RR 0.49, 95% CI 0.31–0.79) and AAD use (RR 0.75, 95% CI 0.61–0.92) were observed to be significantly lower for patients in the early ablation cohort as compared with their counterparts in the late ablation group (Figure 2).

**Table 2:** Outcomes of interest by time interval between AF diagnosis and CA receipt

	Unweighted rate (24-mo event)		*P* value	Weighted rate (24- mo event)∗		*P* value	Unweighted rate (12- mo event)		*P* value	Weighted rate (12- mo event)∗		*P* value
	Early ablation	Late ablation		Early ablation	Late ablation		Early ablation	Late ablation		Early ablation	Late ablation	
Outcome												
Composite outcome	177 (48.10)	166 (62.41)	<.01	49.32	61.39	.01	138 (37.50)	131 (49.25)	<.01	38.46	49.14	.02
AF-related hospitalizations	84 (22.83)	70 (26.32)	.36	22.66	24.55	.61	42 (11.41)	34 (12.78)	.69	11.05	12.49	.60
Electrical cardioversion	43 (11.68)	56 (21.05)	<.01	13.78	19.34	.10	17 (4.62)	25 (9.40)	.03	5.93	8.63	.26
Repeat CA	30 (8.15)	45 (16.92)	<.01	8.56	17.35	<.01	12 (3.26)	29 (10.90)	<.01	3.28	12.08	<.01
AAD use	125 (33.97)	131 (49.25)	<.01	35.95	47.92	.01	109 (29.62)	107 (40.23)	.01	31.36	39.12	.07

Values are n (%) or %. *P* values were obtained by chi-square tests. Early ablation was defined as ablation within 6 months after index AF diagnosis. Late ablation was defined as ablation 6–24 months after the index AF diagnosis.

The first 90 days after CA receipt was blanking period, and events that occurred within this period were not treated as outcome of interest for analysis.

AAD = antiarrhythmic drug; AF = atrial fibrillation; CA = catheter ablation.

∗ Trimming was applied, and we only included patients with propensity score between 0.10 and 0.90 for inverse probability of treatment weighting.

When examining 24-month outcomes by sex, the composite outcome was not observed to be significant for females (RR 0.87, 95% CI 0.66–1.16); however, male patients who had early ablation were observed to have a significantly lower risk of composite outcome as compared with their peers in the late ablation cohort in the 24-month postablation period (RR 0.77, 95% CI 0.62–0.94). No significant difference among the early and late cohorts on individual outcomes over 24 months was observed by sex (Supplementary Table 2).

**Figure 3:**
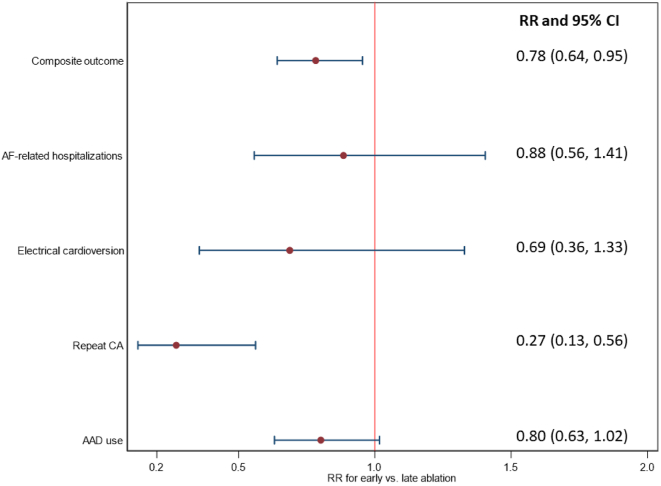
Analysis for association between time to ablation (early vs late) and outcomes within 12 months after catheter ablation (CA). The first 90 days after CA receipt was blanking period, and events that occurred within this period were not treated as the outcome of interest for analysis. Early ablation was defined as ablation within 6 months after index atrial fibrillation (AF) diagnosis. Late ablation was defined as ablation 6–24 months after the index AF diagnosis. AAD = antiarrhythmic drug; CI = confidence interval; RR = risk ratio.

For the 12-month outcome, chi-square tests suggested significantly lower rate of the composite outcome (unweighted: 37.50% vs 49.25%, *P <* .01; weighted: 38.46% vs 49.14%, *P =* .02) and repeat CA (unweighted: 3.26% vs 10.90%, *P <* .01; weighted: 3.28% vs 12.08%, *P <* .01) among the early ablation cohort as compared with the late ablation cohort (Table 2). The unweighted rate of AAD use was significantly lower in the early ablation cohort (unweighted: 29.62% vs 40.23%; *P =* .01), but the rate of AAD use was not significantly different by time to ablation in weighted analysis (weighted: 31.36% vs 39.12%, *P =* .07). Results from the Poisson regression models suggested that patients in early ablation group had a 22% lower risk of composite outcome in the 12-month postablation period than the late ablation group (RR 0.78, 95% CI 0.64–0.95) (Figure 3). No significant difference in AF-related hospitalization (RR 0.88, 95% CI 0.56–1.41), electrical cardioversions (RR 0.69, 95% CI 0.36–1.33), and AAD (RR 0.80, 95% CI 0.63–1.02) was observed for early vs late ablation cohorts. Patients in the early ablation cohort were observed to have significantly lower risk of repeat ablation as compared with those in the late ablation cohort (RR 0.27, 95% CI 0.13–0.56).

## Discussion

Our study suggests that AF patients who received CA within 6 months after AF diagnosis had a significantly lower 24-month risk of AF recurrence than patients receiving CA at a later stage (6–24 months) after AF diagnosis. Risk of repeat CA and AAD use were also significantly lower among the early cohort as compared with the late cohort. Similar results were observed when examining outcomes during the 12-month follow-up period. To the best of our knowledge, this is the first study to examine AF recurrence among patients with AF and HF based on time to ablation since AF diagnosis. The study results suggest that early performance of CA significantly reduces the risk of AF recurrence, primarily evidenced by reduced risk of repeat CA and AAD use, among patients with concomitant AF and HF.

Subgroup analysis by sex indicated that benefits of early ablation to AF recurrence can be less substantial among female patients. Such a differential outcome by sex indicated that female AF patients could be more refractory compared with male patients, which is supported by prior research. For example, a clinical study enrolling 116 AF patients in Australia found that female patients had more advanced atrial remodeling and a higher risk of postablation arrhythmia recurrence than male patients.^27^ Another study, which had 106 Chinese patients with AF, suggested that female biological sex was associated with a 1.2 times higher risk of recurrence after ablation.^28^

A prior study comparing outcomes of CA within 6 months, 6 to 12 months, or 12 to 24 months after diagnosis found similar results. D’Angelo and colleagues^14^ investigated how time to ablation could impact outcomes after CA among AF patients 18 to 64 years of age using the IBM MarketScan Commercial Database. After PS matching by sociodemographic factors and pre-existing health conditions, their results indicated that very early CA (defined as CA within 6 months since diagnosis) was significantly associated with a lower risk of electrical cardioversion than early CA (defined as CA 6–12 months after diagnosis; RR_[very early vs early]_ = 0.74, 95% CI 0.54–0.87) or later CA (defined as CA 12–24 months after diagnosis; RR_[very early vs later]_ = 0.63, 95% CI 0.49–0.79). However, their study population did not contain older patients, and only 12% of them had HF, thereby limiting their generalizability for AF patients with coexisting HF. In a multicenter real-world cohort study in Spain, AF patients (n = 306) receiving ablation within 1 year of diagnosis (vs >1 year) had a 76% lower risk of AF recurrence, which was evidenced by lower rates of postblanking period ablation (4.2% vs 17.9%) and AAD use (26.6% vs 36.3%).^29^ Another retrospective cohort study^15^ used data from the Intermountain Healthcare System in Utah to investigate impact of time to ablation on AF patients by their EF. Results from their regression analysis showed that ablation performed 181 to 545 days after AF diagnosis, compared with 30 to 180 days after AF diagnosis, was associated with a significantly higher 1-year risk of AF recurrence (hazard ratio [HR] 1.42, 95% CI 1.09–1.85) in patients with better heart function (EF >35%) but not in those with EF ≤35% (HR 0.79, 95% CI 0.37–1.72). In addition, patients with HF with preserved EF usually have a better outcome than counterparts with HF with reduced EF. For example, an epidemiologic study containing 28,914 patients found that patients with HF with preserved EF (vs HF with reduced EF) had a lower rate of HF hospitalization (HR 0.72, 95% CI 0.68–0.75), HF-related emergency room visit (HR 0.94, 95% CI 0.90–0.99), and death (HR 0.82, 95% CI 0.79–0.85).^30^ However, the lack of EF in the Optum-SES database precludes the subgroup analysis by EF in the AF patients.

Several studies have shown that following the initial diagnosis of AF, a progression from paroxysmal to persistent and permanent AF occurs.^31^^,^^32^ This progressive nature of AF has been attributed to electrical, contractile, and structural remodeling of the atrium.^33^ Experimental research on animal models has illustrated that AF itself induces electrophysiologic and structural changes within the atria, promoting the perpetuation of AF, often referred to as “AF begets AF.”^33^ Several human studies supported this concept,^34^, ^35^, ^36^ showing that even at a very early stage, in patients with “lone” AF and no underlying cardiovascular disease, atrial structural remodeling changes are already clearly more present.^35^ Consequently, it appears that AF plays a key driver for progressive atrial remodeling. Earlier research has shown that the success rate of AF ablation is lower in cases of more advanced atrial remodeling,^37^^,^^38^ reinforcing the idea that CA should be performed at the earliest possible stage.

Timely CA intervention has the potential to mitigate AF burden among patients with AF and HF. Though not assessed in our study, it is reasonable to assume that lower AF recurrence among patients undergoing early ablation is likely to translate into improved quality of life and lower healthcare costs. Further research could focus on these measures to further highlight the impact of early CA intervention in this population of AF patients with coexisting HF.

### Study limitations

Our study has several limitations. First, the observational nature of this dataset cannot provide strong and causal conclusions of a multicenter, randomized clinical trial design. Second, our reliance on diagnosis codes for AF and heart failure could have influenced the study results. Misclassification could have occurred during the coding process, which might lead to underascertainment of eligible patients; in addition, the underascertainment of the study population could also reflect the study period occurring before some of the relevant randomized controlled trials^39^^,^^40^ showing significant benefit of ablation in AF patients with HF. Third, variables indicating the severity of HF, such as left ventricular EF and New York Heart Association functional class of HF, are unavailable in the Optum claims database. In addition, information on ablation technique and AF severity are also not available. The absence of these variables could impact the study results due to the bias in statistical analysis. Fourth, the assessment of recurrences was symptom driven and not based on systematic rhythm monitoring with heart monitors or implantable loop recorders. As a result, the accuracy of measurement of AF recurrence could have been impacted. Fifth, patients with persistent AF usually have a higher risk of progression and adverse outcomes than those with paroxysmal AF^41^; however, the sample size of persistent AF is low in our study, making it difficult to explore the heterogeneity by AF subtype. Last, the Optum database only includes patients with commercial insurance in the United States, thereby limiting the generalizability of our conclusion.

### Conclusion

This analysis of outcomes after AF ablation in patients with HF and newly diagnosed AF suggests that patients undergoing early CA within 6 months of AF diagnosis have a lower risk of AF recurrence as compared with those who underwent late ablation. The lower risk of AF recurrence was primarily evidenced by a lower risk of repeat ablation and AAD use.
