# Supplementary material for: Influence of time to ablation on outcomes among patients with atrial fibrillation with pre-existing heart failure
Source: Heart Rhythm O2. 2024 Aug 5;5(9):606–13. doi: 10.1016/j.hroo.2024.07.016 (PMC11524955; doi:10.1016/j.hroo.2024.07.016)
Supplement: Supplementary Tables [file mmc1.docx]

**Supplementary Table 1.** Code for study variables

| Variable | Code |
| --- | --- |
| Atrial fibrillation | ICD-9: 427.31  ICD-10: I48.0, I48.1x, I48.2x, I48.91 |
| Heart failure | ICD-9: 39891, 40201, 40211, 40291, 40401, 40403, 40411, 40413, 40491, 40493, 428, 4280, 4281, 4282, 42821, 42822, 42823, 4283, 42831, 42832, 42833, 4284, 42841, 42842, 42843, 4289  ICD-10: I099, I110, I130, I132, I50, I501, I502, I5020, I5021, I5022, I5023, I503, I5030, I5031, I5032, I5033, I504, I5040, I5041, I5042, I5043, I50810, I50811, I50812, I50813, I50814, I5082, I5083, I5084, I5089, I509, P290 |
| Catheter ablation | CPT: 93656  ICD-9: 37.34  ICD-10: 02553ZZ, 02563ZZ, 02573ZZ, 02583ZZ, 025K3ZZ, 025L3ZZ, 025M3ZZ, 025S3ZZ, 025T3ZZ |
| Valvular disease | ICD-9: 093.2x, 394.x–397.x, 424.x,746.01, 746.02, 746.09, 746.00, 746.1, 746.3–746.6, V42.2, V43.3  ICD-10: A52.0, I05.x–I08.x, I09.1, I09.8, I34.x–I39.x, Q23.0–Q23.3, Z95.2–Z95.4 |
| Cardiac arrhythmias | ICD-9: 426.0, 426.13, 426.9, 426.10, 426.11, 426.12, 426.13, 426.2–426.54, 426.6–426.8x, 427.0–427.4, 427.5, 427.6–427.9, 785.0-785.3, 794.31, 996.01, 996.04, V45.0x, V53.3x  ICD-10: I44.1–I44.3, I45.6, I45.9, I47.x–I49.x, R00.0, R00.1, R00.8, T82.1, Z45.0, Z95.0 |
| Pulmonary circulation disorders | ICD-9: 415.0, 415.1x, 416.x, 417.0, 417.8, 417.9  ICD-10: I26.x, I27.x, I28.0, I28.8, I28.9 |
| Peripheral vascular disorders | ICD-9: 093.0, 437.3, 440.x, 441.x, 443.1–443.9, 447.1, 557.1, 557.9, V43.4  ICD-10: I70.x, I71.x, I73.1, I73.8, I73.9, I77.1, I79.0, I79.2, K55.1, K55.8, K55.9, Z95.8, Z95.9 |
| Hypertension, uncomplicated | ICD-9: 401.x  ICD-10: I10.x |
| Hypertension, complicated | ICD-9: 402.x–405.x  ICD-10: I11.x–I13.x, I15.x |
| Paralysis | ICD-9: 334.1, 342.x, 343.x, 344.0–344.6, 344.9  ICD-10: G04.1, G11.4, G80.1, G80.2, G81.x, G82.x, G83.0–G83.4, G83.9 |
| Other neurological disorders | ICD-9: 331.9, 332.0, 332.1, 333.4, 333.5, 333.92, 334.x–335.x, 336.2, 340.x, 341.x, 345.x, 348.1, 348.3, 780.3, 784.3  ICD-10: G10.x–G13.x, G20.x–G22.x, G25.4, G25.5, G31.2, G31.8, G31.9, G32.x, G35.x–G37.x, G40.x, G41.x, G93.1, G93.4, R47.0, R56.x |
| Chronic pulmonary disease | ICD-9: 416.8, 416.9, 490.x –505.x, 506.4, 508.1, 508.8  ICD-10: I27.8, I27.9, J40.x–J47.x, J60.x–J67.x, J68.4, J70.1, J70.3 |
| Diabetes, uncomplicated | ICD-9: 250.0-250.3x, 250.8x, 250.9x  ICD-10: E10.618, E10.620, E10.621, E10.622, E10.628, E10.630, E10.638, E10.649, E10.65, E10.69, E10.8, E10.9, E11.618, E11.620, E11.621, E11.622, E11.628, E11.630, E11.638, E11.649, E11.65, E11.69, E11.8, E11.9, E13.618, E13.620, E13.621, E13.622, E13.628, E13.630, E13.638, E13.649, E13.65, E13.69, E13.8, E13.9 |
| Diabetes, complicated | ICD-9: 250.4x-250.7x  ICD-10: E10.21, E10.22, E10.29, E10.311, E10.319, E10.3211, E10.3212, E10.3213, E10.3219, E10.3291, E10.3292, E10.3293, E10.3299, E10.3311, E10.3312, E10.3313, E10.3319, E10.3391, E10.3392, E10.3393, E10.3399, E10.3411, E10.3412, E10.3413, E10.3419, E10.3491, E10.3492, E10.3493, E10.3499, E10.3511, E10.3512, E10.3513, E10.3519, E10.3521, E10.3522, E10.3523, E10.3529, E10.3531, E10.3532, E10.3533, E10.3539, E10.3541, E10.3542, E10.3543, E10.3549, E10.3551, E10.3552, E10.3553, E10.3559, E10.3591, E10.3592, E10.3593, E10.3599, E10.40, E10.41, E10.42, E10.43, E10.44, E10.49, E10.51, E10.52, E10.59, E10.610, E10.65, E11.21, E11.22, E11.29, E11.311, E11.319, E11.3211, E11.3212, E11.3213, E11.3219, E11.3291, E11.3292, E11.3293, E11.3299, E11.3311, E11.3312, E11.3313, E11.3319, E11.3391, E11.3392, E11.3393, E11.3399, E11.3411, E11.3412, E11.3413, E11.3419, E11.3491, E11.3492, E11.3493, E11.3499, E11.3511, E11.3512, E11.3513, E11.3519, E11.3521, E11.3522, E11.3523, E11.3529, E11.3531, E11.3532, E11.3533, E11.3539, E11.3541, E11.3542, E11.3543, E11.3549, E11.3551, E11.3552, E11.3553, E11.3559, E11.3591, E11.3592, E11.3593, E11.3599, E11.40, E11.41, E11.42, E11.43, E11.44, E11.49, E11.51, E11.52, E11.59, E11.610, E11.65, E13.21, E13.22, E13.29, E13.40, E13.41, E13.42, E13.43, E13.44, E13.49, E13.51, E13.52, E13.59, E13.610 |
| Hypothyroidism | ICD-9: 240.9, 243.x, 244.x, 246.1, 246.8  ICD-10: E00.x–E03.x, E89.0 |
| Renal failure | ICD-9: 403.01, 403.11, 403.91, 404.02, 404.12, 404.92, 404.03, 404.13, 404.93, 582.x, 583.0–583.7, 585.x, 586, 588.0, V42.0, V45.11, V56.x  ICD-10: I12.0, I13.1, N18.x, N19.x, N25.0, Z49.0–Z49.2, Z94.0, Z99.2 |
| Liver disease | ICD-9: 070.22, 070.23, 070.32, 070.33, 070.44, 070.54, 070.6, 070.9, 456.0–456.2, 570.x, 571.x, 572.2–572.8, 573.3, 573.4, 573.8, 573.9, V42.7  ICD-10: B18.x, I85.x, I86.4, I98.2, K70.x, K71.1, K71.3–K71.5, K71.7, K72.x–K74.x, K76.0, K76.2–K76.9, Z94.4 |
| Peptic ulcer disease excluding bleeding | ICD-9: 531.7, 531.9, 532.7, 532.9, 533.7, 533.9, 534.7, 534.9  ICD-10: K25.7, K25.9, K26.7, K26.9, K27.7, K27.9, K28.7, K28.9 |
| AIDS/HIV | ICD-9: 042.x–044.x  ICD-10: B20.x–B22.x, B24.x |
| Lymphoma | ICD-9: 200.x–202.x, 203.0, 238.6  ICD-10: C81.x–C85.x, C88.x, C96.x, C90.0, C90.2 |
| Metastatic cancer | ICD-9: 196.x–199.x  ICD-10: C77.x–C80.x |
| Solid tumor without metastasis | ICD-9: 140.x–172.x, 174.x–195.x  ICD-10: C00.x–C26.x, C30.x–C34.x, C37.x–C41.x, C43.x, C45.x–C58.x, C60.x–C76.x, C97.x |
| Rheumatoid arthritis / collagen vascular diseases | ICD-9: 446.x, 701.0, 710.0–710.4, 710.8, 710.9, 711.2, 714.x, 719.3, 720.x, 725.x, 728.5, 728.89, 729.30  ICD-10: L94.0, L94.1, L94.3, M05.x, M06.x, M08.x, M12.0, M12.3, M30.x, M31.0–M31.3, M32.x–M35.x, M45.x, M46.1, M46.8, M46.9 |
| Coagulopathy | ICD-9: 286.x, 287.1, 287.3–287.5  ICD-10: D65–D68.x, D69.1, D69.3–D69.6 |
| Obesity | ICD-9: V85.30, V85.31, V85.32, V85.33, V85.34, V85.35, V85.36, V85.37, V85.38, V85.39, V85.41, V85.42, V85.43, V85.44, V85.45, 278.00, 278.01, 278.03  ICD-10: Z68.24, Z68.25, Z68.26, Z68.27, Z68.28, Z68.29, Z68.3, Z68.30, Z68.31, Z68.32, Z68.33, Z68.34, Z68.35, Z68.36, Z68.37, Z68.38, Z68.39, Z68.41, Z68.42, Z68.43, Z68.44, Z68.45, E66.9, E66.09, E66.1, E66.8, E66.01, E66.2 |
| Weight loss | ICD-9: 260.x–263.x, 783.2, 799.4  ICD-10: E40.x–E46.x, R63.4, R64 |
| Fluid and electrolyte disorders | ICD-9: 253.6, 276.x  ICD-10: E22.2, E86.x, E87.x |
| Blood loss anemia | ICD-9: 280.0  ICD-10: D50.0 |
| Deficiency anemia | ICD-9: 280.1–280.9, 281.x  ICD-10: D50.8, D50.9, D51.x–D53.x |
| Alcohol abuse | ICD-9: 265.2, 291.1–291.3, 291.5–291.9, 303.0, 303.9, 305.0, 357.5, 425.5, 535.3, 571.0–571.3, 980.x, V11.3  ICD-10: F10, E52, G62.1, I42.6, K29.2, K70.0, K70.3, K70.9, T51.x, Z50.2, Z71.4, Z72.1 |
| Drug abuse | ICD-9: 292.x, 304.x, 305.2–305.9, V65.42  ICD-10: F11.x–F16.x, F18.x, F19.x, Z71.5, Z72.2 |
| Psychoses | ICD-9: 293.8, 295.x, 296.04, 296.14, 296.44, 296.54, 297.x, 298.x  ICD-10: F20.x, F22.x–F25.x, F28.x, F29.x, F30.2, F31.2, F31.5 |
| Depression | ICD-9: 296.2, 296.3, 296.5, 300.4, 309.x, 311  ICD-10: F20.4, F31.3–F31.5, F32.x, F33.x, F34.1, F41.2, F43.2 |
| Stroke/TIA/thromboembolism | ICD-9: 433, 433.01, 433.1, 433.11, 433.2, 433.21, 433.3, 433.31, 433.8, 433.81, 433.9, 433.91, 434, 434.01, 434.1, 434.11, 434.9, 434.91, 435, 435.1, 435.2, 435.3, 435.8, 435.9, 436, 437, 437.1, 437.2, 437.3, 437.4, 437.5, 437.6, 437.7, 437.8, 437.9, 438, 438.1, 438.11, 438.12, 438.13, 438.14, 438.19, 438.2, 438.21, 438.22, 438.3, 438.31, 438.32, 438.4, 438.41, 438.42, 438.5, 438.51, 438.52, 438.53, 438.6, 438.7, 438.81, 438.82, 438.83, 438.84, 438.85, 438.89, 438.9  ICD-10: I65.1, I63.02, I63.12, I63.22, I65.21, I65.22, I65.23, I65.29, I63.031, I63.032, I63.033, I63.039, I63.131, I63.132, I63.133, I63.139, I63.231, I63.232, I63.233, I63.239, I65.01, I65.02, I65.03, I65.09, I63.011, I63.012, I63.013, I63.019, I63.111, I63.112, I63.113, I63.119, I63.211, I63.212, I63.213, I63.219, I65.8, I63.59, I65.8, I63.09, I63.19, I63.59, I65.9, I63.00, I63.10, I63.20, I63.29, I66.01, I66.02, I66.03, I66.09, I66.11, I66.12, I66.13, I66.19, I66.21, I66.22, I66.23, I66.29, I66.3, I66.9, I63.30, I63.311, I63.312, I63.313, I63.319, I63.321, I63.322, I63.323, I63.329, I63.331, I63.332, I63.333, I63.339, I63.341, I63.342, I63.343, I63.349, I63.39, I63.6, I66.01, I66.02, I66.03, I66.09, I66.11, I66.12, I66.13, I66.19, I66.21, I66.22, I66.23, I66.29, I66.3, I66.9, I63.40, I63.411, I63.412, I63.413, I63.419, I63.421, I63.422, I63.423, I63.429, I63.431, I63.432, I63.433, I63.439, I63.441, I63.442, I63.443, I63.449, I63.49, I66.01, I66.02, I66.03, I66.09, I66.11, I66.12, I66.13, I66.19, I66.21, I66.22, I66.23, I66.29, I66.3, I66.8, I66.9, I63.50, I63.511, I63.512, I63.513, I63.519, I63.521, I63.522, I63.523, I63.529, I63.531, I63.532, I63.533, I63.539, I63.541, I63.542, I63.543, I63.549, I63.59, I63.8, I63.9G45.0G45.0G45.8G45.0G45.1G45.2G45.8G46.0G46.1G46.2G45.9, I67.841, I67.848, I67.89, I67.2, I67.81, I67.82, I67.89, I67.4, I67.1, I67.7, I68.2, I67.5, I67.6, G45.4, G46.3, G46.4, G46.5, G46.6, G46.7, G46.8, I67.89, I68.0, I68.8, I67.9, I69.010, I69.011, I69.012, I69.013, I69.014, I69.015, I69.018, I69.019, I69.110, I69.111, I69.112, I69.113, I69.114, I69.115, I69.118, I69.119, I69.210, I69.211, I69.212, I69.213, I69.214, I69.215, I69.218, I69.219, I69.310, I69.311, I69.312, I69.313, I69.314, I69.315, I69.318, I69.319, I69.810, I69.811, I69.812, I69.813, I69.814, I69.815, I69.818, I69.819, I69.910, I69.911, I69.912, I69.913, I69.914, I69.915, I69.918, I69.919, I69.928, I69.020, I69.120, I69.220, I69.320, I69.820, I69.920, I69.021, I69.121, I69.221, I69.321, I69.821, I69.921, I69.022, I69.122, I69.222, I69.322, I69.822, I69.922, I69.023, I69.123, I69.223, I69.323, I69.823, I69.923, I69.028, I69.128, I69.228, I69.328, I69.828, I69.928, I69.059, I69.159, I69.259, I69.359, I69.859, I69.959, I69.051, I69.052, I69.151, I69.152, I69.251, I69.252, I69.351, I69.352, I69.851, I69.852, I69.951, I69.952, I69.053, I69.054, I69.153, I69.154, I69.253, I69.254, I69.353, I69.354, I69.853, I69.854, I69.953, I69.954, I69.039, I69.139, I69.239, I69.339, I69.839, I69.939, I69.031, I69.032, I69.131, I69.132, I69.231, I69.232, I69.331, I69.332, I69.831, I69.832, I69.931, I69.932, I69.033, I69.034, I69.133, I69.134, I69.233, I69.234, I69.333, I69.334, I69.833, I69.834, I69.933, I69.934, I69.049, I69.149, I69.249, I69.349, I69.849, I69.949, I69.041, I69.042, I69.141, I69.142, I69.241, I69.242, I69.341, I69.342, I69.841, I69.842, I69.941, I69.942, I69.043, I69.044, I69.143, I69.144, I69.243, I69.244, I69.343, I69.344, I69.843, I69.844, I69.943, I69.944, I69.069, I69.169, I69.269, I69.369, I69.869, I69.969, I69.061, I69.062, I69.161, I69.162, I69.261, I69.262, I69.361, I69.362, I69.861, I69.862, I69.961, I69.962, I69.063, I69.064, I69.163, I69.164, I69.263, I69.264, I69.363, I69.364, I69.863, I69.864, I69.963, I69.964, I69.065, I69.165, I69.265, I69.365, I69.865, I69.965, I69.998, I69.998, I69.090, I69.190, I69.290, I69.390, I69.890, I69.990, I69.091, I69.191, I69.291, I69.391, I69.891, I69.991, I69.092, I69.192, I69.292, I69.392, I69.892, I69.992, I69.093, I69.193, I69.293, I69.393, I69.893, I69.993, I69.998, I69.098, I69.198, I69.298, I69.398, I69.898, I69.998, I69.00, I69.10, I69.20, I69.30, I69.80, I69.90 |
| Vascular Disease | ICD-9: 410.00, 410.01, 410.02, 410.10, 410.11, 410.12, 410.20, 410.21, 410.22, 410.30, 410.31, 410.32, 410.40, 410.41, 410.42, 410.50, 410.51, 410.52, 410.60, 410.61, 410.62, 410.70, 410.71, 410.72, 410.80, 410.81, 410.82, 410.9, 410.91, 410.92, 411.0, 412, 429.79  ICD-10: I21.09, I21.09, I22.0, I21.09, I21.09, I21.01, I21.02, I21.09, I22.0, I21.09, I21.19, I21.19, I22.1, I21.19, I21.11, I21.11, I22.1, I21.11, I21.19, I21.19, I22.1, I21.19, I21.29, I21.29, I22.8, I21.29, I21.29, I21.29, I22.8, I21.29, I21.4, I21.4, I22.2, I21.4, I21.29, I21.21, I21.29, I22.8, I21.29, I21.9, I21.A1, I21.A9, I21.3, I21.3, I21.9, I21.A9, I21.A1, I22.9, I21.A1, I21.9, I21.A9, I21.3, I24.1, I25.2, I23.0, I23.3, I23.6, I23.7, I23.8 |
| Chest pain | ICD-9: 786.50, 786.51, 786.52, 786.59  ICD-10: R07.1, R07.2, R07.89, R07. 9 |
| Edema | ICD-9: 782.3  ICD-10: R60.0, R60.1, R60.9 |
| Orthopnea | ICD-9: 786.02  ICD-10: R06.01 |
| Ascites | ICD-9: 789.59  ICD-10: R18.8 |
| Sleep apnea | ICD-9: 327.2, 327.21, 327.22, 327.23, 327.24, 327.25, 327.26, 327.27, 327.29  ICD-10: G47.3, G47.30, G47.31, G47.32, G47.33, G47.34, G47.35, G47.36, G47.37, G47.39, P28.3, R06.81 |
| Cardiomyopathy | ICD-9: 4250, 42511, 42518, 4252, 4253, 4254, 4255, 4257, 4258, 4259  ICD-10: I423, I421, I422, I428, I424, I420, I425, I429, I426, I43, A1884, I427 |
| Congenital heart disease | ICD-9: 745-747  ICD-10: Q20-Q28 |
| Cardiac resynchronization therapy defibrillator and/or implantable cardioverter defibrillator | ICD-9: V45.02  ICD-10: Z95810, Z950, 02H40KZ, 02H44KZ, 02H60KZ, 02H63KZ, 02H64KZ, 02H70KZ, 02H73KZ, 02H74KZ, 02HK0KZ, 02HK3KZ, 02HK4KZ, 02HL0KZ, 02HL3KZ, 02HL4KZ, 02HN0KZ, 02HN3KZ, 02HN4KZ, 0JH608Z, 0JH638Z, 0JH808Z, 0JH838Z, 0JH809Z, 0JH839Z, 0JH609Z, 0JH639Z  CPT: 33216, 33217, 33224, 33225, 33230, 33231, 33240, 33245, 33246, 33249, 33270, 33271, 93283, 93284, 93287, 93289, 93295, 93296, 93745  HCPCS: C1882, G0448 |

**Supplementary Table 2.** Comparison of 24-month study outcomes in early versus late CA cohort in female and male patients using Poisson regression (24-month outcomes)

|  | **RR and 95% CI** | |
| --- | --- | --- |
|  | **Female** | **Male** |
| **Composite outcome** | 0.87 (0.66, 1.16) | 0.77 (0.62, 0.94) |
| **AF-related hospitalizations** | 0.85 (0.51, 1.42) | 0.84 (0.54, 1.28) |
| **Electrical cardioversion** | 0.65 (0.31, 1.37) | 0.71 (0.42, 1.19) |
| **Repeat CA** | 0.49 (0.22, 1.08) | 0.63 (0.33, 1.22) |
| **AAD use** | 0.69 (0.48, 0.99) | 0.76 (0.58, 1.00) |

Abbreviations: AAD: antiarrhythmic drug, AF: atrial fibrillation, CA: catheter ablation

The first 90 days after CA receipt was blanking period, and events that occurred within this period were not treated as outcome of interest for analysis.

Early ablation was defined as ablation within 6 months after index AF diagnosis. Late ablation was defined as ablation 6-24 months after the index AF diagnosis.
